# Root separation modulates AMF diversity and composition in tomato–potato onion intercropping systems

**DOI:** 10.3389/fmicb.2025.1554644

**Published:** 2025-03-12

**Authors:** Musawar Ibrahim, Asad Ullah, Xinjie Pan, Jianzeng Lu, Musaddiq Ibrahim, Kunpeng Cao, Shouwei Liu, Xingang Zhou, Fengzhi Wu, Danmei Gao

**Affiliations:** ^1^Key Laboratory of Biology and Genetic Improvement of Horticultural Crops (Northeast Region), Department of Horticulture, Ministry of Agriculture and Rural Affairs, Northeast Agricultural University, Harbin, China; ^2^School of Mathematics and Statistics, Xi’an Jiaotong University, Xi’an, Shaanxi, China; ^3^Department of Mathematics, Air University, Islamabad, Pakistan

**Keywords:** AMF, CMNs, intercropping, root barrier, plant-plant interaction, soil microbial community

## Abstract

Plant-plant interactions shape arbuscular mycorrhizal fungi (AMF) communities in rhizosphere soil, with tomato/potato-onion intercropping emerging as a promising agro-ecological strategy to optimize resource utilization. However, the role of root separation methods in modulating AMF diversity within intercropping systems remains unclear. Specifically, whether the AMF community in the rhizosphere of tomato and potato-onion intercropping differs from monoculture and how root separation methods modulate these effects. This study evaluates the effects of various root separation methods (no separation, 0.45 μm nylon membrane, 38 μm nylon membrane, and solid separation) on AMF diversity and composition in tomato/potato-onion intercropping and monoculture systems. High-throughput Illumina MiSeq sequencing was used to assess AMF diversity indices (Ace, Chao1, Shannon, and Simpson), and Principal Coordinate Analysis evaluated community structure. Results showed that the non-separation mode achieved the highest Ace and Chao1 indices, indicating greater richness, while intercropping lowered Shannon and Simpson indices. Intercropping significantly reduced *Glomerales* but increased *Paraglomerales*, under the non-separation mode. Similarly, it decreased *Glomus* while increasing *Paraglomus* in the rhizosphere of both crops. Principal Coordinate Analysis revealed that root separation distinctly altered AMF community structure, reflecting specific barrier effects on AMF interactions. Intercropping increased AMF abundance in the tomato rhizosphere but reduced it in potato-onion as shown by 18S rRNA gene abundance. These findings emphasize that minimizing root separation in intercropping enhances AMF diversity and functionality, providing valuable insights for sustainable agricultural management. Understanding the role of root interactions in shaping AMF communities can help optimizing intercropping strategies to improve soil health and nutrient dynamics.

## 1 Introduction

In sustainable agriculture, enhancing soil health and beneficial microbial interactions is vital for crop resilience and productivity. Common mycorrhizal networks (CMNs) play a key role in structuring the composition and diversity of arbuscular mycorrhizal fungi (AMF) by facilitating nutrient exchanges and modifying root exudates (Barto et al., 2012). Extending beyond individual root zones, CMNs create shared nutrient spaces, enriching rhizosphere AMF communities with essential nutrients and signaling compounds that support diverse and functional microbial roles (Gupta et al., 2019; Oelmüller, 2019; Ullah et al., 2024). Under nutrient-limited conditions, such as low phosphorus, CMNs can transfer nutrients between plants, supporting AMF species adept at nutrient acquisition. This redistribution may lead to shifts in AMF community composition, as species with efficient nutrient uptake capacities become more dominant in nutrient-poor soils (Zhou et al., 2023). Additionally, CMNs enable the sharing of root exudates, allowing coordinated recruitment and structuring of AMF communities across the network (Muneer et al., 2020; Zhou et al., 2023). Despite the clear influence of CMNs on AMF community composition, the potential mechanisms by which CMNs alter the composition of AMF communities remain unclear. Among the various farming practices, intercropping has emerged as a promising strategy to enhance soil health, optimize nutrient use, and increase biodiversity in crop ecosystems. Compared with monoculture, intercropping provides distinct ecosystem benefits by enhancing the soil microbial environment (Ali et al., 2019b). Recent studies have suggested that intercropping can facilitate AMF diversity by increasing plant-microbe interactions and reducing competition for resources (Zhang et al., 2024). Facilitation in this context refers to the phenomenon whereby existence of one species alters the environment in a manner that improves the fitness of neighboring species (Brooker et al., 2021; Yu et al., 2021). These notions support that microbial communities that are helpful to plants tend to be more abundant and diverse in diversified agro-ecosystems as opposed to communities of plants that are species deficient or species-poor plant communities such as monoculture systems (Garbeva et al., 2006; Latz et al., 2015; Zhou et al., 2017). AMF play a key role in nutrient cycling and energy flow, providing the base for soil health and crop productivity, making it important to understand how cropping systems influence microbial community dynamics (Wang et al., 2018). For example, intercropping-induced shifts in soil AMF communities can modify soil carbon accumulation and nutrient cycling (Sun et al., 2022). Additionally, in intercropping systems with decomposing brassica and cereal residues, the microbial environment in the rhizosphere can be altered, promoting plant resilience against diseases such as tomato bacterial wilt (Gao et al., 2024). Similarly, Ali et al. (2020), found that garlic substrate induces cucumber growth development and decreases Fusarium wilt through regulation of soil microbial community structure and diversity in replanted disturbed soil.

The practice of intercropping diverse plant species can modify the metabolic product of root, organic matter and resource utilization, potentially contributing to the establishment of specialized microbial communities that govern plant growth. Recent studies have also emphasized the root exudation patterns as a key factor in shaping microbial communities, influencing both AMF colonization and plant performance under various environmental conditions (Sharma et al., 2023; Zhang et al., 2024). Moreover, root exudates, which consist of a variety of compounds like amino acids, sugars, and organic acids, can alter the rhizosphere microbiota and enhance the plant’s stress tolerance, providing additional benefits to intercropping systems (Fu et al., 2015; Wu et al., 2023). According to Zhang et al. (2020), intercropping increased soil AMF diversity in the rhizosphere of soybean, whereas no such effect was shown in the neighboring maize. According to Šmilauer et al. (2020), Plant communities could filter AMF or AMF could be driving plant community composition. Observational studies in natural ecosystems cannot differentiate whether AMF diversity supports greater plant diversity or AMF diversity is dependent on plant composition (Lekberg and Waller, 2016; Kokkoris et al., 2020). Understanding whether increasing crop diversity can bolster AMF communities that could benefit sustainable agricultural systems requires a thorough investigation of the underlying mechanisms between crop and AMF diversity.

Tomato and potato-onion intercropping, have gained considerable attention in China as an effective strategy to improve resource utilization and soil health. This intercropping strategy can be best described as companion cropping where both crops interact beneficially, leading to enhanced soil conditions. Gong et al. (2019), found that tomato intercropped with potato-onion significantly altered tomato growth, improved nutrient uptake, and increased the abundance of rhizosphere AMF communities. Previous studies have reported that soil AMF abundance responds differently to intercropping, with asymmetric effects on tomato (*Solanum lycopersicum* L.) and potato-onion (*Allium cepa* var. *aggregatum*) growth. However, the community composition of AMF shifted only in the rhizosphere of potato onion (Houlden et al., 2008; Gao et al., 2021b). Wu et al. (2016), reported that intercropping tomato with potato-onion enhanced tomato growth and phosphorus uptake, increased phosphobacteria diversity, and reduced soil acidification. Shi et al. (2021), observed that volatile organic compounds from potato-onion influenced tomato root morphology, while Yu et al. (2017), found that intercropping potato-onion promoted deeper tomato roots and increased biomass under varying phosphorus levels. Gao et al. (2021b), highlighted the influence of intercropping and phosphorus fertilization on tomato and potato-onion growth, as well as AMF diversity, improving phosphorus availability. Similarly, Li N. et al. (2020), demonstrated that intercropping increased the diversity of bacterial and fungal communities in the tomato rhizosphere, enhancing nutrient uptake. Additionally, He et al. (2021), showed that intercropping with potato-onion, coupled with biochar application, improved tomato growth by regulating soil properties and microbial communities. Although the benefits of intercropping on AMF diversity and soil microbial communities are well documented, the specific influence of root separation methods (such as nylon membranes and solid barriers) on AMF communities in vegetable-based intercropping systems in China remains insufficiently explored.

Permeable barriers typically made of mesh with pore sizes that allow hyphal passage but restrict direct root penetration, are widely used to promote mycorrhizal connectivity without direct root-to-root competition. This method allows for the formation of common mycorrhizal networks (CMNs), enabling nutrient exchange and microbial signaling between plants. Studies have shown that in systems with permeable barriers, AMF diversity is enhanced, leading to increased phosphorus and nitrogen uptake through CMNs, which benefit both plants in the intercropping system (Muneer et al., 2020; Zhou et al., 2023). For example, in a tomato–potato onion intercropping setup, permeable barriers have been observed to promote a disease-suppressive microbiome in the tomato rhizosphere by facilitating *Bacillus* spp. recruitment, which enhances systemic resistance to soil borne pathogens (Zhou et al., 2023). In experiments with semi-permeable mesh, root exudates from companion plants have been shown to stimulate beneficial microbial recruitment in the rhizosphere. For instance, exudates from the potato onion can enhance the recruitment of AMF and promote beneficial bacterial communities, indirectly increasing tomato plant growth and resilience in the intercropping system (Gao et al., 2021b; Li et al., 2022). In cases of tomato and potato-onion intercropping with solid partitions, the root exudates from potato-onion, notably secondary metabolites like taxifolin, can still enhance the recruitment of beneficial microbes, albeit with reduced efficiency compared to systems with permeable barriers (Zhou et al., 2023). Understanding how root separation methods (no separation, 0.45 μm and 38 μm nylon membranes, solid partitioning) affect AMF community composition and diversity is essential to uncovering the mechanisms by which AMF contribute to intercropping benefits. These techniques, which restrict root-to-root competition while maintaining nutrient exchange via CMNs, may influence the abundance and diversity of AMF communities in these systems (Zhou et al., 2023). Recent studies indicate that root separation methods can alter microbial diversity, but their influence on AMF communities, especially in tomato and potato-onion intercropping, remains unclear.

This study addresses these knowledge gaps by evaluating AMF diversity and community composition in tomato and potato-onion intercropping under different root separation methods.

We hypothesize that (1) AMF community abundance and diversity will be higher in intercropping systems compared to monoculture due to increased resource diversity and plant-microbe interactions. (2) Different root separation methods will differentially affect AMF communities, with no separations promoting greater AMF diversity and abundance than partial separation or complete separation. To test these hypotheses, we used high-throughput Illumina MiSeq sequencing to assess AMF communities across treatments. The findings of this study will provide critical insights into how root separation influences AMF functionality and its potential ecological relevance for sustainable agriculture, with a focus on optimizing intercropping systems in China.

## 2 Materials and methods

### 2.1 Study area and soil description

The greenhouse experiment was carried out at Horticulture Experimental Station of Northeast Agricultural University, Harbin, China (45°41′N, 126°37′E). Tomato (cv. Dongnong 708), and potato onion (var. *Suihua*) were used as plant material. P-deficient soil was chosen because AMF establish CMNs more effectively under low phosphorus conditions. The P-deficient soil taken from 10 cm to 15 cm of depth of an open field in Northeast Agricultural University was used in this experiment. To ensure consistency across treatments, all pots used the same homogenized soil batch before planting. This approach minimizes potential confounding factors that could arise from variations in soil composition. The baseline physicochemical properties of the soil were as follows: organic matter content of 2.68%, inorganic nitrogen levels (NH_4_^–^-N and NO_3_^–^-N) at 121.67 mg kg^–1^, available phosphorus (AP) at 10.32 mg kg^–1^, available potassium (AK) at 70.19 mg kg^–1^, electrical conductivity (EC) at 0.25 mS cm^–1^, and a pH of 6.61. These properties were consistent across all pots, ensuring uniformity and eliminating soil history or microbial legacy effects as sources of variation in AMF diversity.

### 2.2 Experimental setup

**FIGURE 1:**
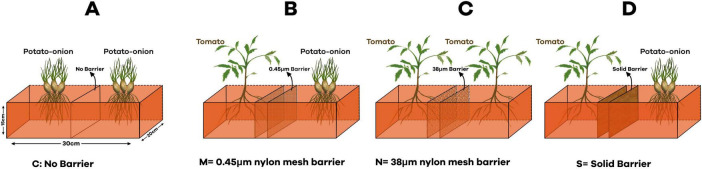
Schematic diagram of experimental design in different barrier treatments. **(A)** No barrier (C); **(B)** 0.45 μm nylon mesh barrier (M); **(C)** 38 μm nylon mesh barrier (N); **(D)** Solid barrier (S).

**FIGURE 2:**
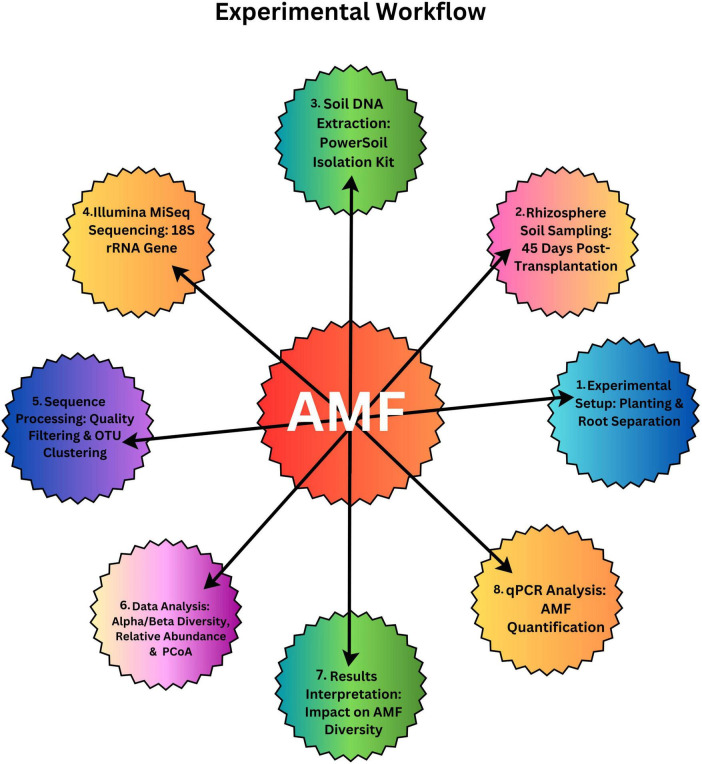
Experimental workflow for the methodology of AMF (Arbuscular Mycorrhizal Fungi) research.

In order to find out the belowground interaction between tomato and potato onion, plastic pots (30 cm × 20 cm × 15 cm), each filled with 3 kg of soil, were separated with four different barriers: (1) no barrier (C) (proper belowground contact) (2) 0.45 μm (M) nylon mesh barrier (no root and AMF contact) (3) 38 μm (N) nylon mesh barrier (no root contact but AMF can pass through) and (4) solid barrier (no belowground contact) (Figure 1). The experimental workflow, which is presented in Figure 2, outlines the entire experimental setup and procedure followed to assess the impact of these treatments on AMF communities. The 0.45 μm and 38 μm nylon mesh barriers were obtained from Sigma-Aldrich, made of high-quality nylon, ensuring permeability for fungal hyphae while preventing root penetration. The solid barriers used were made of PVC, providing complete separation between root systems to isolate plant interactions. There were four cropping patterns: mono-cropped potato-onion (O), intercropped potato-onion and tomato (OT), mono-cropped tomato (T), intercropped tomato and potato-onion (TO). The experiment was designed as a randomized complete block design, each cropping pattern consists of 6 pots with three replicates for individual barrier treatment across different cropping patterns. Therefore, there were [4 barrier treatments (C, M, N and S) × 4 Cropping Patterns (O, OT, T and TO) × 3 replicates × 6 microcosms with total of (4 × 4 × 6 × 3) = 288 pots].

### 2.3 Seedling germination

To prepare the seeds for germinating in a peat: perlite (1:1 v/v) mixture and eventually growing in the experimental greenhouse, tomato seeds were first surface sterilized using 3.8% sodium hypochlorite for 10 min. After that, they were washed three times using sterile distilled water to remove any residue. In tomato/potato-onion intercropping system, the planting ratio for both OT and TO treatment was 1:3, where each pot contained one tomato seedling and three potato-onion bulbs. This planting ratio was selected based on previous studies demonstrating optimal intercropping benefits, including enhanced tomato growth, improved phosphorus uptake, promoted root interactions, and increased microbial diversity in the rhizosphere (Wu et al., 2016; Li N. et al., 2020; Gao et al., 2021a). Half of the pots were used to plant tomato seedlings with two cotyledons, while the other half was used to plant three bulbs of potato onion, spaced 5 cm–10 cm apart. Two tomato seedlings per pot were used in each pot in (T) monoculture cultivation. Half of the pots were used to plant single tomato seedling with two cotyledons and the other half side also planted with a single tomato seedling. Six potato-onion bulbs per pot were used in each pot in (O) monoculture cultivation. Half of the pots were used to plant three potato-onion bulbs and the other half side also planted with three potato-onion bulbs. The greenhouse conditions were maintained as follows: temperature at 25°C–28°C (day) and 18°C–22°C (night), light intensity at 400–600 μmol⋅m^–2^⋅s^–1^ photosynthetically active radiation (PAR), a photoperiod of 14 h light/10 h dark, and relative humidity at 60%–70%. Plants were irrigated with a unique drip irrigation followed the method of Shah and Chu (2021). Soil moisture was maintained at 70%–80% of field capacity using a drip irrigation system, with irrigation applied every two days based on soil moisture measurements using tensiometers and gravimetric methods. The seed company’s recommendations for insect management, weed suppression, and fertilization were all followed.

### 2.4 Rhizosphere soil sampling

Soil samples were collected from both the monoculture and intercropping systems 45 days after transplantation, as CMNs are generally established around 40 days, offering an optimal timeframe for assessing AMF diversity and plant-microbe interactions. After brushing off the roots of each treatment, a sample of the rhizosphere soil that had been attached to the plants was taken (Gao et al., 2021b). For each treatment, three separate samples of plants and rhizosphere soils were taken. Then, from each of these samples, a composite sample was made. As a result, every treatment had three composite samples. To extract DNA, soil samples from the rhizosphere were kept at a temperature of −80°C.

### 2.5 Soil DNA extraction

Total soil DNA was extracted from 0.25 g of cucumber rhizosphere soil using the Power Soil DNA Isolation Kit (MO BIO Laboratories, Carlsbad, CA, USA) according to the manufacturer’s protocol. Each composite soil DNA sample was extracted three times, and the three samples were mixed to make composite DNA samples. The DNA purity was measured in spectrophotometer and then stored at −80°C for further analysis.

### 2.6 Quantitative PCR

Total AMF abundances of the soil samples were estimated in triplicate using qPCR assays on an IQ5 real-time PCR system (Bio-Rad Laboratories, Hercules, CA, USA). An 18S rRNA gene fragment was amplified using the primer set AMV4.5NF/AMDGR (Lee et al., 2008). The qPCR reaction mixture was prepared in a total volume of 20 μL, containing 9 μL of 2 × Real SYBR Green Master Mix, 0.5 μL of each primer (10 μM), 2 μL of template DNA, and 8 μL of ddH_2_O. The reaction conditions were as follows: an initial denaturation step at 95°C for 3 min, followed by 32 amplification cycles consisting of 95°C for 30 s (denaturation) and 55°C for 30 s (annealing). A negative control was included, using sterilized water instead of soil DNA. All composite soil samples were analyzed in triplicate. Standard curves for AMF were generated using a 10-fold dilution series of plasmids containing the target genes from soil samples. The amplification efficiency of the AMF standard curve was 99.39%, with an *R*^2^ value of 0.999. The initial copy numbers of AMF target genes were determined by comparing the threshold cycle (Ct) values of the samples to the standard curve.

### 2.7 Illumina MiSeq sequencing

Illumina MiSeq sequencing was used to examine the composition of the rhizosphere AMF community. An 18S rRNA gene fragment was amplified using the primer sets AMV4.5NF/AMDGR (Lee et al., 2008). In the first round of PCR, the primers AML1 and AML2 were utilized. Then, in the second round of PCR, which was appropriate for Illumina MiSeq sequencing, the result was re-amplified using the primers of AMV4.5NF/AMDGR. Each sample was able to be multiplexed using the 7 bp barcodes that were part of the last primer pair in order to do Illumina sequencing. The first round of PCR produced amplicons around 800 bp in size, while the second round produced amplicons around 300 bp in size. In a 20 μL reaction, which included 4 μL of 5 × FastPfu buffer, 2 μL of 2.5 mm dNTPs, 0.8 μL of each primer (5 μM), 10 ng of template DNA, 0.4 μL of FastPfu polymerase (from Transgene Biotech in Beijing, China), and replenishing ddH_2_O to a volume of 20 μL, PCR amplifications were performed. For the first round of PCR, the conditions were 95°C for 3 min. Then, for the second round, the circumstances were 30 cycles of 95°C for 30 s, 55°C for 30 s, and 72°C for 45 s. Finally, there was an extension of 10 min at 72°C. The AxyPrep PCR Clean-up Kit (Oxygen Biosciences, CA, USA) was used to independently amplify each composite soil sample, pool the triplicate results, and purify them. Afterward, equimolar amounts of purified amplicons were pooled and measured using a TBS-380 micro fluorometer using Picogreen reagent (Invitrogen, USA). Following the instructions provided by the manufacturer (Major Bio-Pharm Technology Co. Ltd., Shanghai, China), the amplicon libraries were then sequenced using an Illumina MiSeq Genome Sequencer PE300 Titanium platform.

### 2.8 Raw sequence data processing

Raw sequence reads obtained from the Illumina MiSeq sequencing run were initially de-multiplexed, quality filtered, and processed using the FLASH algorithm (Magoč and Salzberg, 2011). Sequences with poor quality (average quality score < 25) and those shorter than 200 base pairs were discarded to ensure high data quality. Singleton sequences were also removed prior to clustering to minimize their potential impact on diversity estimates (Kunin et al., 2010). Subsequently, the remaining high-quality sequences were clustered into Operational Taxonomic Units (OTUs) at a 97% sequence similarity threshold using the UPARSE algorithm (Edgar, 2013). OTUs that did not belong to the *Glomeromycota* or potentially represented chimeric sequences were excluded from further analysis. Taxonomic assignments of the representative sequences were performed by performing BLAST searches against the Silva 124 database (Chen et al., 2017) and the MaarjAM database (Öpik et al., 2010). The BLAST search required a match to meet the following criteria: sequence coverage ≥ 90%, sequence similarity ≥ 97%, and a BLAST e-value of < 1e-50 (Öpik et al., 2009). All raw sequence reads have been deposited in the NCBI Sequence Read Archive with the submission accession number PRJNA1197118.

### 2.9 Statistics analysis

Alpha diversity indices, including Shannon, Ace, Chao1, and Simpson indices, were calculated using QIIME. Differences in AMF community composition between treatment groups were evaluated through the analysis of similarity (ANOSIM) in QIIME, with further analysis conducted in R (v. 3.4.1) using the “vegan” package. Principal Coordinate Analysis (PCoA) based on Bray-Curtis dissimilarity was used to assess beta diversity, highlighting structural differences in AMF communities across treatments. Quantitative data, including AMF abundance, relative abundance, and alpha diversity indices, were analyzed using IBM SPSS Statistics 21.0. Statistical significance was determined using analysis of variance (ANOVA) at *P* < 0.05, with Tukey’s test applied for pairwise comparisons. All indicator values were determined using R. Software by applying the IndVal method, which calculates the indicator value for each species based on its relative abundance and frequency in each treatment group. A significance threshold of *P* < 0.05 was used to identify species with significant indicator values. Graphical work, including the creation of bar plots and principal coordinate analysis (PCoA) plots, was designed using Origin 2024b. The software was used to customize visual elements such as axis labels, legends, and color schemes to enhance clarity and presentation.

## 3 Results

### 3.1 Effects of different root separation modes on the relative abundance of AMF in the rhizosphere of mono-and intercropping systems

**FIGURE 3:**
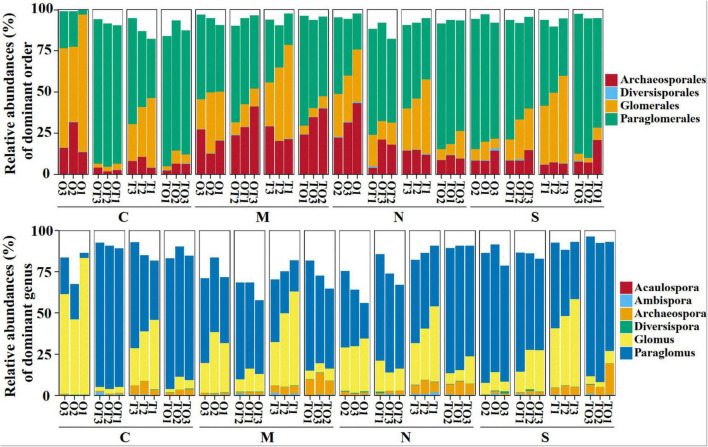
Effects of different root separation methods on the relative abundance of AMF community under order and genus levels in the rhizosphere of mono- and intercropping systems. C, M, N, S represent no separation treatment; 0.45 μm nylon membrane separation treatment; 38 μm nylon membrane separation treatment; solid separation treatment, respectively. O, OT, T, TO: monocropped potato-onion treatment; intercropped potato-onion treatment; monocropped tomato treatment; intercropped tomato treatment, respectively (*P* < 0.05).

**FIGURE 4:**
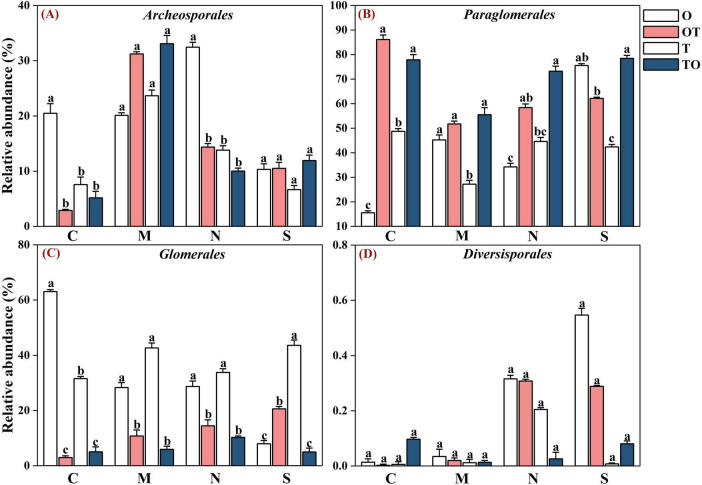
Effects of different root separation methods on the relative abundance of AMF dominant orders in the rhizosphere of mono-and intercropping systems. Panels **(A–D)** represent the relative abundance of *Archeosporales*, *Paraglomales*, *Glomerales*, and *Diversisporales*, respectively. C, M, N, S represent no separation treatment; 0.45 μm nylon membrane separation treatment; 38 μm nylon membrane separation treatment; solid separation treatment, respectively. O, OT, T, TO: monocropped potato-onion treatment; intercropped potato-onion treatment; monocropped tomato treatment; intercropped tomato treatment, respectively. Lowercase letters indicate the significant difference between different planting patterns under the same separation method (*P* < 0.05).

**FIGURE 5:**
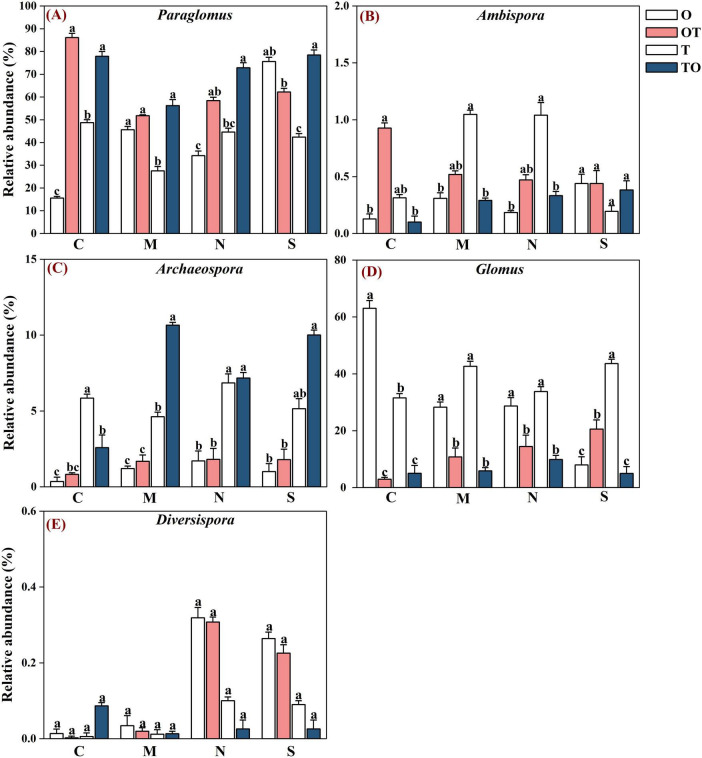
Effects of different root system separation methods on the relative abundance of dominant genera in the rhizosphere of mono-and intercropping systems. Panels **(A–E)** represent the relative abundance of *Paraglomus*, *Ambispora*, *Archaeospora*, *Glomus* and *Diversispora* respectively. C, M, N, S represent no separation treatment; 0.45 μm nylon membrane separation treatment; 38 μm nylon membrane separation treatment; Solid separation treatment, respectively. O, OT, T, TO: monocropped potato-onion treatment; intercropped potato-onion treatment; monocropped tomato treatment; intercropped tomato treatment, respectively. Lowercase letters indicate the significant difference between different planting patterns under the same separation method (*P* < 0.05).

Across all soil samples, Illumina MiSeq sequencing generated 794,562 high quality AMF sequences. The number of sequences in each sample was 33,030–41,901 with the mean of 37,386. The average length of all quality sequences was 216 bp. This graph shows the relative abundances of different AMF orders (*Archaeosporales*, *Diversisporales*, *Glomerales*, and *Paraglomerales*) in the rhizosphere under different root separation treatments. The MiSeq high-throughput sequencing data was classified at 97% similarity level. The AMF community, belonging to the phylum *Glomeromycota* and class *Glomeromycetes*, was categorized into four orders: *Glomerales*, *Paraglomerales*, *Diversisporales*, and *Archaeosporales*. Among these, *Paraglomerales* had the highest relative abundance, followed by *Glomerales* and *Archaeosporales*, with *Diversisporales* showing the lowest relative abundance (Figures 3, 4). Our results demonstrate the influence of different root separation methods (C, M, N, S) on the composition of AMF communities at the order levels. The analysis revealed significant variations in the relative abundances of various AMF orders across the different treatments. Specifically, certain root separation methods were found to selectively favor specific AMF orders. In the non-separation mode, intercropping led to a notable decrease in the relative abundance of *Glomerales* in the rhizosphere of both tomatoes and potato-onions. Conversely, the same intercropping approach in the non-separation mode resulted in a significant increase in the relative abundance of *Paraglomerales* in the rhizospheres of both plants (*P* < 0.05). Additionally, intercropping in non-separation mode significantly reduced the relative abundance of *Glomus* in the rhizosphere of tomatoes and potato-onions, while it significantly increasing the relative abundances of *Paraglomus* in the rhizosphere of these intercropped plants (*P* < 0.05) (Figure 5).

#### 3.1.1 Indicator genera of AMF based on IndVal analysis

**FIGURE 6:**
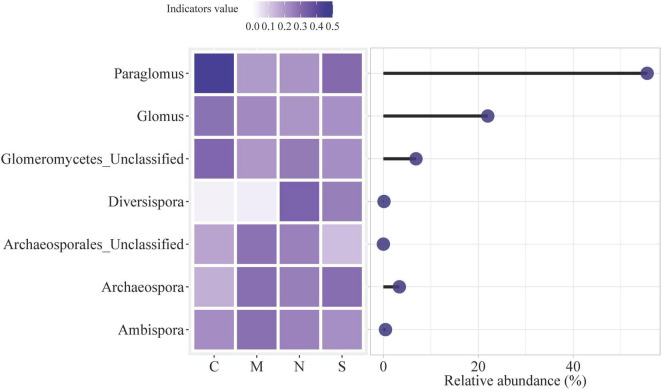
Indicator genera of AMF based on IndVal analysis. Genera with an indicator value > 0.4 and *p* < 0.05 were identified as indicators. The heatmap shows the relative abundances of these AMF taxa in different treatments: no separation (C), 0.45 μm nylon mesh (M), 38 μm nylon mesh (N), and solid barrier (S).

The relative abundance and indicator values of various AMF taxa under different root separation treatments: no separation (C), 0.45 μm nylon mesh (M), 38 μm nylon mesh (N), and solid barrier (S) (Figure 6). The heatmap on the left side indicates the indicator values, which range from 0.0 to 0.5 and are color-coded from light purple (low value) to dark purple (high value). The indicator value reflects how strongly each AMF taxon is associated with a particular root separation method. Darker shades indicate a stronger association, while lighter shades suggest a weaker or less significant association. The circles on the right side of the figure correspond to the relative abundance of these AMF taxa, with larger circles indicating higher relative abundance. The length of the black line extending from each circle represents the range of relative abundance for each taxon across the different treatments. For example, *Paraglomus* shows a high relative abundance in the no separation treatment (C) compared to other taxa, and its abundance decreases with barrier treatments such as M, N and S.

#### 3.1.2 Effect of various root separation methods on AMF diversity indices in tomato and potato-onion systems

**FIGURE 7:**
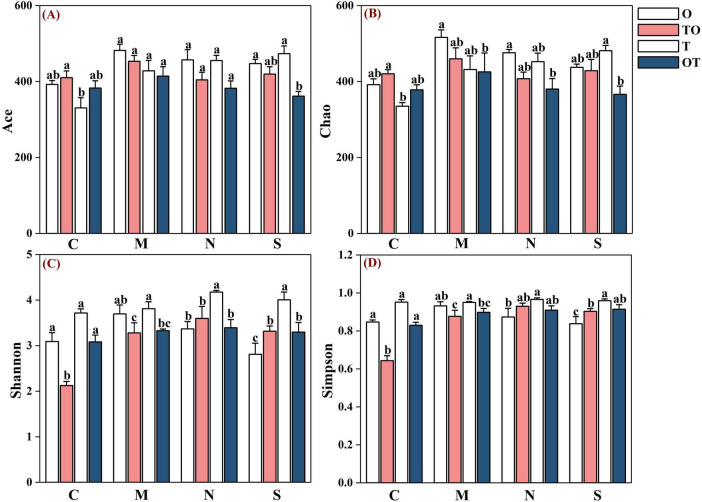
Effects of different root separation modes on the a-diversity index of rhizosphere AMF communities in mono-and intercropping systems. Panels **(A–D)** represent the relative abundance of Ace, Chao, Shannon, and Simpson respectively. C, M, N, S represent no separation treatment; 0.45 μm nylon membrane separation treatment; 38 μm nylon membrane separation treatment; Solid separation treatment, respectively. O, OT, T, TO: monocropped potato-onion treatment; intercropped potato-onion treatment; monocropped tomato treatment; intercropped tomato treatment, respectively. Lowercase letters indicate the significant difference between different planting patterns under the same separation method (*P* < 0.05).

In the non-separation mode, intercropping (both TO and OT systems) resulted in significantly higher richness as indicated by the increased Ace and Chao1 indices, compared to the monocropping tomato system (T) (*P* < 0.05). However, the increase in richness was not statistically different when compared to the monocropping potato-onion system (O). In contrast, intercropping (TO) led to a significant decrease in both the Shannon and Simpson indices (*P* < 0.05), indicating a reduction in species evenness, when compared to both monocropping systems (T and O). Nevertheless, intercropping (OT) resulted in a decrease in both the Shannon and Simpson indices compared to monocropping (T and O), but the differences were not statistically significant. Under the 38 μm separation mode, intercropping resulted in a reduction of the Chao1, Ace, and Shannon indices in both the tomato and potato-onion rhizosphere. However, the results were statistically similar for the Ace index across both systems. This suggests that while certain diversity measures are affected by root separation, others remain stable despite varying treatments. Similarly, under the solid partitioning mode, intercropping resulted in a decrease in all diversity indices in the rhizosphere of both plant systems (Figure 7).

**FIGURE 8:**
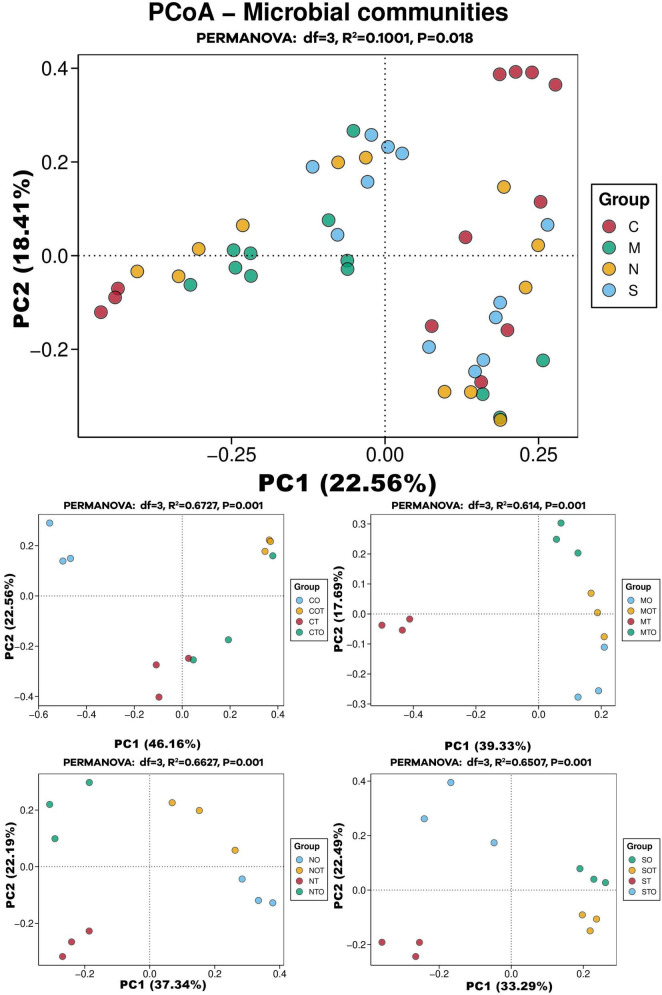
PCoA analysis of AMF community structure in the rhizosphere of mono- and intercropping tomato/potato-onion under different root separation methods. C, M, N, S represent no separation treatment; 0.45 μm nylon membrane separation treatment; 38 μm nylon membrane separation treatment; Solid separation treatment, respectively. O, OT, T, TO: monocropped potato-onion treatment; intercropped potato-onion treatment; monocropped tomato treatment; intercropped tomato treatment, respectively (*P* < 0.05).

In order to analyze the effects of different separation methods on the changes in AMF community structure in the rhizosphere of tomato intercropped with potato-onions, the sequencing data were flattened according to the minimum sequence number among all samples and then analyzed by PCoA (Figure 8). The three replicates of a single treatment clustered together in the PCoA map, indicating good repeatability and consistency in AMF community structure across biological replicates for each treatment. This experiment was based on PCoA mapping based on the Bray-Curtis dissimilarity distance. It can be seen from the table that there are obvious differences in the rhizosphere AMF communities of different planting patterns under different separation methods (Table 1).

**TABLE 1:** PCoA significance analysis of AMF community structure in the rhizosphere of tomato intercropped with potato-onion plants under different root system separation methods.

Categories	Adonis		ANOSIM		MRPP		
	***R* ^2^**	***P***	***R***	***P***	**Delta**	**Effect size**	***P***
All	0.687	0.001	0.825	0.001	0.648	0.327	0.001
C	0.672	0.001	0.778	0.001	0.705	0.342	0.001
M	0.614	0.001	0.790	0.001	0.607	0.256	0.001
N	0.662	0.001	0.957	0.001	0.617	0.314	0.001
S	0.651	0.001	0.926	0.001	0.584	0.307	0.002

C, M, N, S represent no separation treatment; 0.45 μm nylon membrane separation treatment; 38 μm nylon membrane separation treatment; Solid separation treatment, respectively (*P* < 0.05).

#### 3.1.3 Effects of different root separation methods on the abundance of AMF communities in the rhizosphere of tomato and potato-onion systems

**FIGURE 9:**
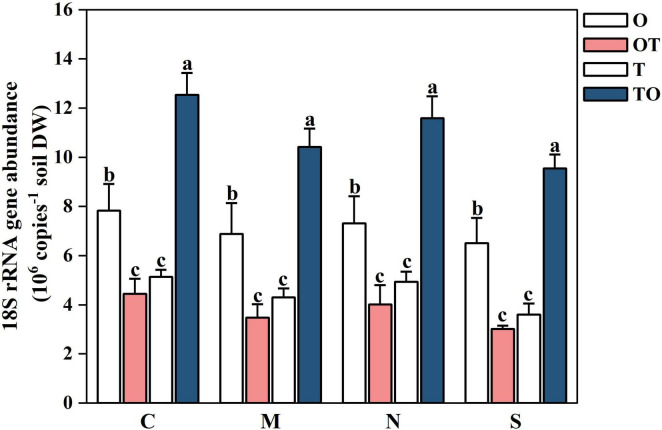
Effects of different root system separation methods on the abundance of AMF communities in the rhizosphere of mono-and intercropping systems. C, M, N, S represent no separation treatment; 0.45 μm nylon membrane separation treatment; 38 μm nylon membrane separation treatment; Solid separation treatment, respectively. O, OT, T, TO: monocropped potato-onion treatment; intercropped potato-onion treatment; monocropped tomato treatment; intercropped tomato treatment, respectively. Lowercase letters indicate the significant difference between different planting patterns under the same separation method (*P* < 0.05).

Under different root separation methods, intercropping increased the AMF community abundance in the tomato rhizosphere, but decreased the AMF community abundance in the potato-onion rhizosphere (*P* < 0.05). The reduction in AMF abundance under restrictive root separation conditions is likely due to both restricted fungal growth and reduced root-microbe interactions. Root separation hinders the formation of CMNs, which are essential for AMF colonization and nutrient exchange between plants. The effects of root separation were consistent across both intercropping systems (TO and OT), although a stronger reduction in AMF abundance was observed in the potato-onion rhizosphere compared to the tomato rhizosphere. This suggests that the tomato system may be more resilient to root separation, maintaining higher AMF abundance despite the physical barriers. The figure clearly illustrates the influence of root system separation on AMF communities, showing that intercropping (TO and OT) consistently enhanced AMF abundance relative to monoculture systems (O and T), with the tomato intercropping treatment (TO) showing the highest AMF abundance across all separation methods (*P* < 0.05) (Figure 9). In contrast monoculture systems particularly single tomato (T) and single potato-onion (O) treatments, exhibit lower AMF abundance, with the differences being more pronounced under restrictive separation conditions. The separation methods further influence AMF abundance, where no separation (C) supports the highest AMF abundance across all planting systems, followed by decreasing levels of abundance under 38 μm membrane separation (N), 0.45 μm membrane separation (M), while the lowest abundance was found in solid separation (S). This decrease indicates that physical and chemical interactions between root systems are critical for sustaining AMF communities.

## 4 Discussion

The first hypothesis, which proposed that AMF diversity and abundance would be higher in intercropping systems compared to monoculture, was partially supported by the data. Intercropping systems led to promote higher AMF richness, as indicated by the increased Ace and Chao1 indices. However, contrary to expectations, AMF community evenness, as indicated by the Shannon and Simpson indices, was lower in intercropped systems. This suggests that while intercropping may promote a more diverse range of AMF species, it might also lead to a more uneven distribution of these species, potentially due to competition or altered ecological interactions between the plants in the system. This finding aligns with previous studies that suggest intercropping can lead to both increased species diversity and shifts in community structure, where some species may dominate, thus reducing overall evenness (Sun et al., 2022).

The second hypothesis, which predicted that no root separation would enhance AMF diversity and abundance compared to partial or complete separation, was largely supported. In the non-separation mode, we observed an increase in AMF richness, consistent with the idea that unrestricted root interactions promote microbial diversity by providing a wider variety of ecological niches. However, root separation specifically using 38 μm and 0.45 μm mesh barriers reduced AMF diversity, particularly at the genus level. This suggests that root separation methods hinder the exchange of microbial communities, limiting the potential for AMF to colonize across plant systems. This finding aligns with previous studies suggest that complete separation, by restricting plant-microbe interactions, likely impedes the dynamic development of AMF communities, while the non-separation mode allows for more flexible microbial exchanges and the establishment of diverse AMF communities (Khashi u Rahman et al., 2021).

Root separation techniques in intercropping systems have been shown to significantly influence the composition and diversity of AMF communities. This shift is driven by several interrelated factors, including the disruption of CMNs, alterations in root exudate diffusion, and changes in the spatial and functional niches available to AMF species. A comprehensive mechanistic explanation, focusing on how these factors contribute to AMF community dynamics, follows.

CMNs can connect plants of either the same or different species and facilitate the long-distance transfer of essential nutrients, including nitrogen, phosphorus, potassium, carbon, and other micronutrients (Walder et al., 2012; Teste et al., 2015; Whiteside et al., 2019; Muneer et al., 2020; Gao et al., 2021a). When root systems are separated, as in the case of using physical barriers like nylon membranes, the formation of CMNs is disrupted, preventing fungal hyphae from linking the rhizosphere of different plants (Weremijewicz and Janos, 2019; Muneer et al., 2020). The absence of CMNs in root-separated systems results in reduced AMF diversity, as network-mediated nutrient transfer is impaired. In intercropping systems, plants frequently rely on shared fungal networks for nutrient acquisition and mutualistic benefits (Gao et al., 2021a). Without CMNs, plants may not be able to exchange nutrients efficiently, leading to isolated fungal communities (Figueiredo et al., 2021). This disruption explains the observed reduction in AMF diversity and the alteration of community composition in root-separated systems.

Root exudates are another critical factor influencing AMF community dynamics. Plants release a wide range of chemical compounds into the rhizosphere soil, including sugars, amino acids, organic acids, and phenolic compounds, which act as signals for AMF communities (Tahat and Sijam, 2012; Rasmann and Turlings, 2016; Lyu and Smith, 2022; Lone et al., 2024). These exudates can attract beneficial microorganisms, or inhibit pathogens, depending on their chemical composition. In root-separated conditions, the diffusion of these exudates between roots is restricted, leading to changes in the recruitment of AMF species. In such conditions, AMF may not have access to the full spectrum of exudate compounds that would typically support a diverse community. In non-separation mode, root exudates and nutrient exchange via CMNs help maintain a more uniform chemical environment, allowing a greater diversity of AMF species. In contrast, root separation limits fungal access to the shared nutrient pool by disrupting CMNs and restricting the movement of exudates, leading to the formation of distinct micro-niches within the rhizosphere (Weremijewicz and Janos, 2019; Muneer et al., 2020; Gao et al., 2021a). These micro-niches are characterized by unique nutrient profiles and chemical cues, which may favor specific AMF species adapted to those particular conditions. For instance, AMF species specialized for phosphorus acquisition may dominate zones with higher concentrations of organic acids, while other species may thrive in areas with elevated levels of sugars or amino acids (Andrino et al., 2021). This niche partitioning process further drives changes in AMF community composition, as species that are more competitive for specific nutrients or chemical cues will dominate in their respective root zones.

Our finding indicates that the non-separation mode of intercropping conditions, the relative abundance of *Glomerales* in the rhizospheres of both tomatoes and potato-onions showed a significant decrease. Conversely, the same intercropping approach in the non-separation mode led to a significant increase in the relative abundance of *Paraglomerales* in the rhizospheres of both plants. The non-separation mode in intercropping, where plant roots are in close contact and share a common rhizosphere, leads to a decrease in *Glomerales* and an increase in *Paraglomerales*. This shift can be attributed to the diversification of root exudates, root competition, plant community composition, and the ability of *Paraglomerales* to colonize a broader range of plant species (Tisserant et al., 2013; Jiang et al., 2018; Guo et al., 2021; Lang et al., 2022). *Glomerales* are commonly found in more homogeneous root environments, such as monocultures, where the root exudates of a single plant species are more consistent and provide a stable environment for these fungi (Tedersoo et al., 2013). However, in intercropping systems, the presence of multiple plant species leads to root exudate diversification, which can be less favorable for the growth of *Glomerales* (Parihar et al., 2020). Furthermore, competition for soil resources like nutrients and space further diminishes the dominance of *Glomerales* (Koide and Peoples, 2013). In contrast, *Paraglomerales*, being more generalist, can exploit a wider variety of root exudates and are better suited to complex and competitive rhizospheres (Tedersoo et al., 2013). The increased nutrient exchange efficiency of *Paraglomerales* and their ability to colonize a broader range of plant roots make them more adaptable in intercropping systems (Khashi u Rahman et al., 2021). Intercropping systems create diverse root niches, favoring *Paraglomerales* colonization and increasing AMF diversity (Berruti et al., 2016). Thus, *Paraglomerales* thrive in the heterogeneous environments of intercropping, where their broad ecological niche allows them to exploit diverse root exudates and cope with resource competition.

Additionally, the non-separation mode of intercropping significantly alters the composition of AMF communities, reducing the abundance of *Glomus* and promoting *Paraglomus*. This ecological shift is driven by diverse root exudates, resource competition, and variations in plant traits such as phenology and root architecture (Maherali and Klironomos, 2007; Davison et al., 2016). Similarly, Ali et al. (2021), highlighted how various cropping systems regulate microbial diversity and metabolic capabilities, showing that AMF diversity influences overall soil microbial community structure and function. Evidence from Mwakilili et al. (2021), supports this, showing that *Glomus* was more enriched in monoculture maize plots compared to intercropping systems. The relationship between root exudates and AMF colonization is further illustrated by the role of photosynthetic carbon fixation pathways, particularly involving aspartic acid, which has been linked to the formation of AMF. Agnihotri et al. (2021), highlighted the strong correlation between aspartic acid and the relative abundance of *Glomus*, indicating that changes in root exudate chemistry directly influence AMF composition. This suggests that differences in exudate profiles, as seen in intercropping, may disrupt the specific pathways required for *Glomus* colonization. Meanwhile, *Paraglomus* dominates in systems with diverse root exudates, as found in intercropping and crop rotation, due to its greater ecological flexibility (Wachowska and Rychcik, 2023). Mwakilili et al. (2021), also observed increased *Paraglomus* abundance under intercropping, while *Glomus* remained dominant in monocropping. The genus *Paraglomus* has significant implications for plant growth and nutrient uptake in intercropping systems. It is widely recognized for its role in improving nitrogen and phosphorus (P) uptake by plants, which is particularly beneficial in P-deficient soils (Suchitwarasan, 2020; Etesami et al., 2021). This is especially relevant in intercropping systems, where phosphorus availability is often a limiting factor for plant growth. The differential trends observed between *Paraglomus* and *Glomus* in intercropping systems are likely attributed to their unique ecological roles and functional adaptations to specific environmental conditions. The observed shift toward *Paraglomus* in tomato/potato-onion intercropping systems may explain the improved nutrient uptake, including enhanced phosphorus and nitrogen acquisition, which subsequently supports plant growth.

However, some studies, such as Furze et al. (2017), suggest that intercropping does not always result in predictable shifts in AMF diversity or composition, emphasizing the need for further research on the context-dependent effects of intercropping on AMF communities’ conditions under which intercropping can predictably shift AMF communities. In the non-separation mode, intercropping increased the Ace and Chao1 indices, suggesting a higher richness of microbial species in the rhizosphere of both plant systems. These results align with previous studies that have shown that intercropping often promotes microbial diversity by creating diverse root exudate profiles and offering a variety of ecological niches for soil microorganisms (Bender et al., 2016). The increased root biomass and varied chemical signals in intercropping systems are thought to promote a greater number of microbial species, particularly those involved in nutrient cycling and disease suppression (Ren et al., 2024). However, despite the increase in richness, both the Shannon and Simpson diversity indices were lower in the intercropped systems. This suggests that while species richness increased, the evenness of microbial populations decreased, possibly reflecting the dominance of certain microbial taxa over others (Sun et al., 2022). Such a shift in community structure, where a few species become more abundant while others are suppressed, has been observed in intercropping systems and can be attributed to competitive or facilitative interactions between plant species or microbial groups (Lian et al., 2018). The dominance of specific microbial groups might reflect plant species’ different root exudates or the selection pressures imposed by plant-microbe interactions (Zhang et al., 2024). In contrast, under the 38 μm separation modes, intercropping led to a reduction in microbial richness, as indicated by the decreased Chao1, Ace, and Shannon indices. This suggests that root separation experiments often demonstrate that AMF diversity and abundance can be influenced by the degree of root contact between intercropped species. Reduced contact, as in 38 μm separation, may lead to decline microbial exchange and hence AMF diversity. AMF often require hyphal connections to spread across different root systems. A 38 μm partition prevents these larger hyphae from crossing, limiting AMF colonization to specific zones, reducing the overall diversity (Chao1, Ace) (Rillig and Mummey, 2006). This reduction in microbial diversity under partitioning is further consistent with studies showing that spatial separation can limit the overlap of root exudates and reduce microbial dispersal between plant systems (Liu et al., 2022). The physical barriers likely prevent the synergistic microbial interactions that are often observed in non-separation intercropping systems, such as nutrient sharing, cross-feeding, and the exchange of beneficial microorganisms (Khashi u Rahman et al., 2021). Similarly, Ali et al. (2019a), demonstrated that AMF can interact synergistically with organic amendments to enhance nutrient cycling and support plant growth, suggesting that root separation could impact these interactions by limiting fungal and microbial contact. The most striking result was observed under the solid partitioning mode, where intercropping resulted in a decrease in all diversity indices across both tomato and potato-onion rhizosphere. This complete separation (S) likely created a more restrictive environment for microbial dispersal, leading to isolated, less diverse microbial communities within each plant’s rhizosphere (Li C. et al., 2020). The decrease in microbial diversity under the solid separation mode also suggests that the degree of physical separation has a strong influence on the microbial community’s structure and function. While intercropping is generally associated with an increase in microbial diversity and ecosystem functions, the partitioning of the rhizosphere at such block scales may counteract these benefits by limiting microbial colonization, reducing nutrient sharing, and preventing beneficial plant-microbe interactions (Bender et al., 2016; Wang et al., 2018).

The clustering observed in the PCoA plots shows that different planting patterns (e.g., monoculture vs. intercropping) result in significantly different AMF community compositions in the rhizosphere. This is similar to previous findings that showed AMF communities can differ drastically based on plant species and planting systems. A study by Jansa et al. (2019), observed that intercropping altered the composition of AMF communities compared to monoculture systems, suggesting that plant interactions influence AMF diversity. The separation of roots (through non-separation, 0.45 μm, 38 μm and solid separation) in the current study has a clear impact on the diversity and structure of rhizosphere AMF communities. This has been observed in studies where physical barriers between plant roots were found to influence the sharing of AMF networks due to restricted fungal hyphae exchange between plants (Gao et al., 2021b). The clear separation in the PCoA plots based on these treatments reflects the importance of planting patterns and root separation in shaping rhizosphere AMF communities, aligning well with previous studies on AMF community responses to intercropping, nutrient availability, and root compartmentalization (Khashi u Rahman et al., 2021).

The qPCR results suggest that root separation and intercropping significantly impact the abundance of AMF community. The abundance of AMF community in the tomato rhizosphere had been increased by intercropping under different root separation treatments, but it decreased in the potato-onion rhizosphere. The observed variations correspond with prior research suggesting that growth and nutrient consumption of tomato plants, as well as alterations in the rhizosphere AMF community abundance, proceeded when tomato were intercropped with potato-onion (Wu et al., 2016; Gao et al., 2021b).

The impact of root separation on AMF community structure is likely to vary under different environmental conditions such as soil type, climate, and crop species. For example, in soils with low fertility or phosphorus deficiency, the role of AMF becomes even more critical for nutrient uptake. Root separation in such environments could have a more pronounced effect on AMF communities by limiting their ability to extend their hyphal networks and access nutrients. On the other hand, in soils with high fertility or adequate nutrient levels, the impact of root separation might be less severe, as plants may rely less on AMF for nutrient acquisition (Suchitwarasan, 2020; Etesami et al., 2021). High temperatures or extreme moisture levels may also affect AMF colonization and their symbiotic relationships with plants, potentially altering how root separation impacts the AMF community (Jamiołkowska et al., 2018). Similarly, the type of crop species used in the intercropping system could also influence the outcome. Some crops may release specific root exudates that promote AMF. Given the potential variability of root separation effects under different conditions, future studies should investigate how different soil types, climate conditions, and crop species influence AMF community composition and function. Research could explore the interactions between these variables and their collective impact on AMF-mediated soil health benefits, including nutrient cycling, disease resistance, and plant growth promotion. Understanding these interactions would provide a more comprehensive view of how root separation practices can be optimized for sustainable agricultural systems.

## 5 Conclusion

This research specifically highlights the role of root separation methods in modulating AMF diversity and composition within tomato/potato-onion mono and intercropping systems. Our findings reveal that the non-separation treatment promotes higher AMF richness, evidenced by increased Ace and Chao1 indices, and shifts the AMF community toward *Paraglomerales* and *Paraglomus* species, which are associated with improved nutrient dynamics. Previous studies suggested that AMF play a vital role in optimizing nutrient uptake and plant growth in intercropping systems, and our results confirm that root separation significantly influences AMF community structure and functionality. These findings address this scientific question of how root separation impacts AMF diversity and mediates plant-microbe interactions, demonstrating its importance in optimizing intercropping systems. From a practical perspective, avoiding physical barriers between intercropped plants enhances AMF diversity and functionality, facilitating better nutrient exchange and plant growth. Optimizing plant spacing ensures effective root interaction and reduces competition, whereas applying organic amendments such as compost or organic fertilizers further supports AMF colonization and improves phosphorus and nitrogen availability. Additionally, reducing soil disturbance through minimal tillage helps preserve AMF networks, promoting long-term soil fertility and microbial activity, while integrating rotational intercropping maintains AMF diversity and prevents soil degradation. These findings provide valuable insights into the mechanisms regulating AMF interactions, offering pathways to enhance agricultural productivity and sustainability. By implementing these strategies, farmers can harness the benefits of AMF-enhanced intercropping to improve soil health, nutrient uptake, and overall crop resilience. Further exploration is required to understand how root separation influences AMF functional roles at the molecular level, unlocking strategies to improve intercropping efficiency and soil health.

## Data Availability

The original contributions presented in this study are publicly available. This data can be found here: https://www.ncbi.nlm.nih.gov/, accession number PRJNA1197118.
